# Asymptotic Normality and Convergence Rates for Tsallis Entropy Estimators via Stabilization Techniques

**DOI:** 10.3390/e28060619

**Published:** 2026-05-31

**Authors:** Mehmet Sıddık Çadırcı, Martin Singull

**Affiliations:** 1 Department of Statistics, Faculty of Science, Cumhuriyet University, 58140 Sivas, Türkiye; msiddikcadirci@cumhuriyet.edu.tr; 2 Department of Mathematics, Linköping University, 581 83 Linköping, Sweden

**Keywords:** Tsallis entropy, nearest-neighbor estimator, stabilization, Poisson point process, binomial point process, normal approximation, convergence rate

## Abstract

We study nearest-neighbor-based estimators of Tsallis entropy associated with Poisson and binomial point processes on general metric measure spaces. In this study, by combining existing stabilization methods with the validation of the estimator’s local *k*-nearest-neighbor structure, we investigate nearest-neighbor-based Tsallis entropy estimators under Poisson and binomial distributed input data. Rather than proposing a new second-order Poincaré inequality, this paper details and clearly presents stabilization-based normal approximation bounds for Tsallis-type *k*-NN functionals. We establish asymptotic normality and derive explicit convergence rates for the Kolmogorov distance. Our analysis avoids explicit score-function decompositions and instead relies on flexible localizations of add-one costs, which simplify the treatment of higher-order terms. Under natural stabilization and moment conditions, the resulting bounds recover the classical normal approximation rates $s^{-1/2}$ and $n^{-1/2}$ and extend corresponding results for Shannon and Rényi entropy estimators. We further illustrate the scope of the framework through examples involving Tsallis entropy functionals, weighted *k*-NN Shannon entropy estimators. The examples provided highlight the benefits of stabilization-based normal approximations for non-parametric statistical inference in complex spatial and high-dimensional settings.

## 1. Introduction

Let (X,F,d,Q) be a metric measure space, where *Q* is a σ-finite measure and d:X×X→[0,∞) is a metric. We represent $P_{s}$ as a Poisson point process on X with density measure $\lambda_{s}=sQ$, with s≥1. When *Q* is a probability measure, let us also consider the binomial point process $\xi_{n}=\sum_{i=1}^{n}\delta_{X_{i}}$; here, $X_{1},\ldots ,X_{n}$ denote independent observations with joint distribution *Q*. This paper examines the asymptotic distributional properties of nearest-neighbor Tsallis entropy estimators based on these point processes and derives quantitative normal approximation limits for the associated stabilizing functionals.

Entropy and associated information measures play a central role in statistics, information theory, machine learning, and the analysis of complex systems [1,2]. This paper aims to develop a new framework for studying non-extensive entropy measures. Many systems of practical interest, such as those encountered in finance, hydrology, turbulence, and networked dynamical systems, exhibit non-extensive behavior that is poorly captured by purely logarithmic entropy measures. Tsallis entropy [3] is a parametric family of generalized entropies that lies between heavy-tailed and compactly supported regimes and has become a standard tool for studying non-equilibrium and long-range-dependent phenomena. New developments in machine learning provide additional motivation to explore generalized entropy measures beyond the classical Shannon framework. In the fields of reinforcement learning and human-guided reinforcement learning (RLHF), entropy-based regularization, which aims to control exploration, prevent early policy convergence, and mitigate over-optimization, is widely used. Furthermore, modern reward and loss distributions can exhibit heavy-tailed behavior; in such cases, classical KL-based information-theoretic tools may be less effective. Recently, studies on the limits of tail-aware information theory for RLHF and stochastic gradient Langevin dynamics have highlighted the importance of heavy-tailed rewards and losses in such settings [4]. In parallel, the Tsallis entropy framework is used to generalize maximum entropy reinforcement learning by introducing an entropic index that controls the exploration behavior of the learned policy [5]. Although this paper does not directly examine RLHF, these developments further encourage the statistical analysis of Tsallis-type entropy estimators under flexible distribution assumptions. Methodologically, nearest neighbor-based estimators form a flexible class of non-parametric entropy estimators that do not involve direct density estimation. Regarding Shannon entropy, this series of studies includes the Kozachenko–Leonenko estimator and its improvements [6], whereas for generalized entropies such as Rényi and Tsallis, several *k*-NN structures are proposed and evaluated empirically. Specifically, Tsallis-based goodness-of-fit tests using *k*-NN estimators are developed in [7]; there, the emphasis is on test statistics for multivariate generalized Gaussian and *q*-Gaussian distributions. Entropy-based tests for generalized Gaussian distributions based on Shannon entropy and the maximum entropy principle have been investigated in [8]. Supplementary Rényi-based goodness-of-fit tests are proposed in [9] for multivariate Student and Pearson type II distributions, based on the principles of maximum Rényi entropy and nearest neighbor Rényi entropy estimators.

Although these studies highlight the practical potential of Shannon, Tsallis, and Rényi entropy estimators for goodness-of-fit testing, their focus is primarily on consistency, mean-square convergence, and calibration of critical values via Monte Carlo methods, as well as consistency for a class of Rényi entropy estimators, examining the empirical behavior of the corresponding test statistics. At the same time, Refs. [7,8] present extensive simulation results for entropy-based tests under generalized Gaussian and related models.

The complex local dependency structure of nearest neighbor-based functionals creates a significant technical challenge by complicating the application of standard central limit theorems. It was first proposed in [10] for geometric functionals of random point sets and further developed in [11,12,13,14]; stabilization methods provide a powerful framework for capturing local dependence. In recent work, combining stabilization ideas with Malliavin–Stein methods and second-order Poincaré inequalities yields approximation bounds for a wide class of Poisson and binomial point processes [15,16,17,18,19]. Together, these advances have led to sharp central limit theorems for geometric statistics, such as the volumes, face counts, and Betti numbers of random complexes, as well as for functionals related to random graphs.

Contributions and relation to existing work. This paper does not present a new second-order Poincaré inequality or a new general stabilization theorem. Instead, the paper’s contribution lies in adapting existing stabilization and Malliavin–Stein normal approximation tools to the nearest-neighbor Tsallis entropy estimate and in clearly outlining the necessary assumptions and error bounds within this framework. First, we reformulate the Poisson and binomial normal approximation limits using a representation adapted to the nearest-neighbor entropy functionals. The parameters $\Gamma_{1},\ldots ,\Gamma_{6}$ and $\Theta_{K,s},\Theta_{K,n}$ are explicitly defined; thus, the resulting bounds can be directly controlled via “add-one” and second-order “add-one” costs. Second, we verify the basic stabilizing components of the Tsallis *k*-NN estimator under standard density assumptions. Specifically, it is shown that the local *k*-NN score has exponential stabilization properties and satisfies the required uniform $L^{p}$-moment control for compact support, bounded density away from zero and infinity, fixed *k*, fixed entropy index α, and the acceptability condition $p{\left(1-\alpha \right)}_{+}<k$ that it satisfies the required uniform $L^{p}$-moment control. Third, to clarify the statistical role of the entropy index α, we derive the estimator from the following identity:

$$T_{\alpha }\left(f\right)=\frac{1}{1-\alpha }\left(E\{f{\left(X\right)}^{\alpha -1}\}-1\right),$$

We derive the estimator and discuss its relationship with bias, consistency, and the existing nearest-neighbor Shannon, Rényi, and Tsallis entropy estimators. Finally, we briefly discuss how this same stabilization perspective relates to weighted *k*-NN Shannon entropy estimators, Euler characteristic functionals, and minimum spanning tree statistics. The purpose of these examples is to illustrate the scope of the methodology rather than to present new general stabilization results for each case. Therefore, the innovative aspect of this paper is not to replace existing stabilization theory, but to present a validation method specific to the Tsallis model and to provide a transparent, standard approach for estimating nearest-neighbor entropy.

Relation to goodness-of-fit tests. The current results provide a probabilistic complement to entropy-based goodness-of-fit tests for generalized Gaussian and related models. In the study [8], the authors developed *k*-NN Shannon entropy estimators and tests derived from maximum entropy principles based on generalized Gaussian families, obtained the $L^{2}$-consistency of the entropy estimator, and performed extensive simulations on both empirical dimension and power. In [7], Tsallis entropy is employed to generate goodness-of-fit statistics for multivariate generalized Gaussian and *q*-Gaussian models, revealing that the Tsallis-based estimator performs effectively in scenarios involving heavy tails and non-Gaussian distributions. The Rényi-based procedures in [9] employ nearest-neighbor Rényi entropy estimators and maximum entropy characterizations for multivariate Student and Pearson type II distributions. Our work provides normal approximation results with explicit bounds for Tsallis and associated entropy estimators, providing an abstract probability basis that can be utilized to motivate Gaussian methods and to guide calibration of critical values in such test problems beyond bootstrap or fully simulation-based methods.

The remainder of the paper is organized as follows. Section 2 presents the basic concepts of point processes, incremental operators, and stabilization, and formulates the basic assumptions employed throughout the paper. Section 3 provides general normal approximation outcomes for Poisson and binomial inputs, accompanied by a clear bound outcome. Section 4 extends these results by applying Tsallis entropy estimators and corresponding geometric statistics; we also summarize a simulation strategy and typical summary statistics. Section 5 provides proofs for the principal theorems and results. Section 6 provides a brief overview of potential extensions.

## 2. Preliminary Information

### 2.1. Notation

For this article, $P_{s}$ indicates a Poisson point process with intensity measure $\lambda_{s}=sQ$, while $\xi_{n}$ represents a binomial point process consisting of *n* independent observations with joint distribution *Q*. The general Poisson and binomial functionals are denoted by $F_{s}$ and $F_{n}$, respectively. The nearest-neighbor Tsallis entropy estimators are designated separately as ${\hat{T}}_{\alpha ,s}$ and ${\hat{T}}_{\alpha ,n}$; here, α∈(0,∞)∖{1} is the constant Tsallis entropy index. The constants $c_{Q}$ and $\beta_{Q}$ have been chosen for the metric growth condition on *Q*. In this section, the fundamental point process framework is recalled, the increment and cost operators are described, and the stabilization and moment assumptions underlying our main results are stated. Keeping the presentation concise, We emphasize the elements necessary for establishing stabilization and the resulting normal approach limits.

### 2.2. Point Processes and Functionals

Suppose X,F,d,Q is a metric measure space with a σ-finite measure *Q*. Let X be equipped with the smallest σ-algebra that makes the following mappings

(1) $$m_{A}:N\to N\cup \left\{0,\infty \right\},\;m_{A}\left(M\right)=M\left(A\right),$$

measurable for every A∈F. For a point process η on X, η takes values in N. We denote the space of η’s square integrable measurable functionals satisfying the condition $E[F{\left(\eta \right)}^{2}]<\infty$ by F:N→R as $L_{\eta }^{2}\left(X\right)$.

**Definition** **1** (Poisson point process [20])**.** *A Poisson point process P(λ) on X having intensity measure λ satisfies the following conditions:*

*1.*  *Given any measurable set B∈F, the random variable P(λ)(B) exhibits a Poisson distribution having parameter λ(B).*
*2.*  *The random variables $P\left(\lambda \right)\left(B_{1}\right),\ldots ,P\left(\lambda \right)\left(B_{m}\right)$ are independent for pairwise disjoint sets $B_{1},\ldots ,B_{m}\in F$.*

**Definition** **2** (Binomial point process [21])**.** *Consider a probability measure Q on X,F and n∈N. We define the binomial point process $\xi_{n}$ as follows:*

(2) $$\xi_{n}=\sum_{i=1}^{n}\delta_{X_{i}},$$

*where $X_{1},\ldots ,X_{n}$ follow an i.i.d. Q law, and $\delta_{x}$ represents the Dirac measure at x∈X.*

For two real-valued random variables *Y* and *Z*, the Kolmogorov measure is defined as

(3) $$d_{K}\left(Y,Z\right)=\underset{t\in R}{\sup}\left|P(Y\leq t)-P(Z\leq t)\right|.$$

Moreover, we will measure this distance to assess convergence to the standard normal limit.

### 2.3. Increment Operators and Stabilization

Methods of stabilization are based on the behavior of functionals under local modifications to the base-point configuration. Increment operators formalize this idea.

**Definition** **3** (Increment operators [16])**.** *Suppose F:N→R is a measurable functional and η is a point process on X. For x∈X, the (first-order) increment operators are as follows:*

(4) $$\Delta_{x}F\left(\eta \right):=F\left(\eta \cup \left\{x\right\}\right)-F\left(\eta \right).$$

*For different x,y∈X, the second-order increment operator can be expressed as*

$$\Delta_{x,y}F\left(\eta \right):=F\left(\eta \cup \left\{x,y\right\}\right)-F\left(\eta \cup \left\{x\right\}\right)-F\left(\eta \cup \left\{y\right\}\right)+F\left(\eta \right).$$

We call a function stable if it becomes insensitive to large changes in the configuration.

**Definition** **4** (Score-based stability [12])**.** *Consider a measurable score function f:X×N→R. Then, for x∈X, f is said to be stabilized at x if, for every finite set $A\subset X\setminus B_{x}\left(R_{x}\right)$, there exists an almost surely finite random radius $R_{x}>0$ such that*

(5) $$f\left(x,(\eta \cap B_{x}\left(R_{x}\right))\cup A\right)=f\left(x,\eta \cap B_{x}\left(R_{x}\right)\right),$$

*where $B_{x}\left(R_{x}\right)$ is the ball of radius $R_{x}$ centered at x. Score-based stabilization can be useful when it can be written as the sum of the local contributions of the functional. For the present work, it is convenient to rely on the strong stabilization properties expressed directly in terms of $\Delta_{x}F$ and $\Delta_{x,y}F$, which are more natural for nearest neighbor-based entropy estimators.*

**Proposition** **1.** *Suppose $F_{s}\left(P_{s}\right)=\sum_{x\in P_{s}}f_{s}\left(x,P_{s}\right)$ is a functional of a Poisson process $P_{s}$ having an intensity of sQ. Then, for x∈X,*

(6) $$\Delta_{x}F_{s}\left(P_{s}\right)=f_{s}\left(x,P_{s}\cup \left\{x\right\}\right)+\sum_{y\in P_{s}}\Delta_{x}f_{s}\left(y,P_{s}\right).$$

*A similar identity stands for functionals of binomial point processes.*

**Remark** **1.** *The strong stabilization of $F_{s}$ suggests that score-based stabilization is reasonable for a reasonable choice of scores; however, the reverse may not hold. Working directly with $\Delta_{x}F_{s}$ and $\Delta_{x,y}F_{s}$ avoids explicit score decompositions and is often more robust for complex functionals such as Tsallis entropy estimators.*

### 2.4. Assumptions

In this section, the main structural assumptions on the underlying space and the stabilizing functionals considered are stated, adapted from [16,18,22].

**Assumption** **1** (Regularity of *Q*)**.** *We assume the measure Q on (X,F) satisfies a condition of regular growth: there are constants $c_{Q}>0$ and $\beta_{Q}>1$ such that, for all x∈X, all r>0, and sufficiently small ϵ>0,*

$$\underset{\epsilon \to 0^{+}}{lim sup}\frac{Q\left(B_{x}\left(r+\epsilon \right)\right)-Q\left(B_{x}\left(r\right)\right)}{\epsilon }\leq c_{Q}\beta_{Q}r^{\beta_{Q}-1}.$$

*Particularly, Q has a diffuse property in the sense that Q({x})=0 for all x∈X; see [16] [Lemma 5.1(a)].*

**Assumption** **2** (Tail bound on stabilization radius.)**.** *Assume that $F_{s}$ is a strongly stabilizing function of $P_{s}$ having a stabilization radius $R_{x}$ at the point x. Assume there exist constants $C_{1},C_{2},\gamma >0$ with the following inequality for all r≥0:*

$$P\left(R_{x}\geq r\right)\leq C_{1}\exp\left(-C_{2}{\left(s^{1/\beta_{Q}}r\right)}^{\gamma }\right).$$

*For binomial processes $\xi_{n}$, a similar bound is assumed using n instead of s.*

**Assumption** **3** (Exponential spatial decay of add-one costs.)**.** *Let K⊆X be a measurable set representing the active region of the functional $F_{s}$. For x,y∈X, define*

$$d_{s}\left(x,K\right):=\underset{z\in K}{\inf}s^{1/\beta_{Q}}d\left(x,z\right),\;d_{s}\left(x,y\right):=s^{1/\beta_{Q}}d\left(x,y\right).$$

*Assume that there exist constants $C_{3},C_{4},\delta >0$ and a number p≥5 such that, for all s≥1 and all x,y∈X,*

(7) $$\begin{array}{cc} {∥\Delta_{x}F_{s}∥}_{L^{p}} & \leq C_{3}\exp\left(-C_{4}d_{s}{\left(x,K\right)}^{\delta }\right), \end{array}$$

(8) $$\begin{array}{cc} {∥\Delta_{x,y}F_{s}∥}_{L^{p}} & \leq C_{3}\exp\left(-C_{4}\max{\left\{d_{s}\left(x,y\right),d_{s}\left(x,K\right),d_{s}\left(y,K\right)\right\}}^{\delta }\right). \end{array}$$

*The corresponding binomial version is obtained by replacing s with n and $P_{s}$ with $\xi_{n}$.*

**Assumption** **4** (Uniform moment condition.)**.** *Let p≥5 be as in Assumption 3. Assume that there exists a constant $M_{p}<\infty$ such that*

(9) $$\underset{s\geq 1}{\sup}\underset{x,y\in X}{\sup}E\left[|\Delta_{x}F_{s}{|}^{p}+{\left|\Delta_{x,y}F_{s}\right|}^{p}\right]\leq M_{p}.$$

*The same condition is assumed for the binomial functionals $F_{n}$, with n replacing s.*

**Remark** **2.** *Assumption 3 is not a tail bound with respect to a threshold variable u. Rather, it is a spatial localization condition controlling the size of the first- and second-order add-one costs as the inserted points move away from the active region K. Magnitude control of the add-one costs is handled through the uniform $L^{p}$-moment condition in Assumption 4, while the exponential factors in (7) and (8) quantify the spatial decay required for the stabilization-based normal approximation bounds.*

**Remark** **3.** *These assumptions have been standard in the stabilization literature and are satisfied in a wide variety of geometric and topological settings; see [16,18,22]. Within the present context, these assumptions are tailored to ensure that the Tsallis entropy estimators considered in Section 4 allow exponential stabilization radius tails and sufficient moment control for the add-one costs, resulting in explicit normal approximation rates.*

## 3. Main Results

In this section, we present the normal approximation bounds for stabilizing functionals of Poisson and binomial point processes. Here, *N* represents a standard normal random variable.

### 3.1. Poisson Input

Suppose $F\in L_{P_{s}}^{2}\left(X\right)$ is a square-integrable functional of a Poisson point process $P_{s}$ having intensity λ=sQ. Given a measurable subset $A_{x}\subseteq X$ depending on *x*, the following are defined:

$$\begin{array}{cc} b_{1}\left(x,A_{x}\right) & :=E{\left|\Delta_{x}F-\Delta_{x}F\left(A_{x}\right)\right|}^{4},\\ b_{2}\left(x,A_{x}\right) & :=E{\left|\Delta_{x}F\left(A_{x}\right)\right|}^{4}. \end{array}$$

For a measurable subset $A_{x}\subset X$, we define the localized add-one cost by

$$\Delta_{x}F\left(A_{x}\right):=F\left(\left(\eta \cap A_{x}\right)\cup \left\{x\right\}\right)-F\left(\eta \cap A_{x}\right).$$

Given a Poisson functional *F*,

$$\sigma_{F}^{2}:=\mathrm{Var}\left(F\right)>0,\;\lambda_{s}:=sQ,$$

let us define

$$A_{p}\left(x\right):=∥\Delta_{x}{F∥}_{L^{p}},\;B_{p}\left(x,y\right):={∥\Delta_{x,y}F∥}_{L^{p}}.$$

Quantities appearing in the Poisson normal approximation limit can be written as follows: $\Gamma_{i}=\Gamma_{i}\left(F,\lambda_{s}\right)$, i=1,…,6. They are the normalized first- and second-order Malliavin–Stein error terms related to the add-one and second-order add-one cost operators. Specifically, we note that they depend only on $\sigma_{F}^{2}$, $A_{p}\left(x\right)$, $B_{p}\left(x,y\right)$, and the density measure $\lambda_{s}$.

**Theorem** **1** (Normal approximation under Poisson input)**.** *Let F be as above and assume that*

(10) $$E\left[F^{2}\right]<\infty \;and\;E\left[\int_{X}{\left(\Delta_{x}F\right)}^{2}\;\lambda \left(dx\right)\right]<\infty .$$

*Then there is an absolute constant $C^{*}>0$ such as*

(11) $$d_{K}\left(\frac{F-E[F]}{\sqrt{\mathrm{Var}(F)}},N\right)\leq \sum_{i=1}^{6}\Gamma_{i,s}\left(F\right),$$

*where the quantities $\Gamma_{1,s}\left(F\right),\ldots ,\Gamma_{6,s}\left(F\right)$ are the Malliavin–Stein error terms defined explicitly in Appendix A. These quantities are expressed in terms of the first-order difference operator $D_{x}F$, the second-order difference operator $D_{x,y}^{2}F$, the variance Var(F), and the intensity measure $\lambda_{s}=sQ$.*

**Remark** **4.** *Theorem 1 uses adaptable addition cost operators to localize increases without requiring a clear point decomposition. It can be seen as a modified improvement of the second-order Poincaré inequality in [15] and the boundary conditions in [16], which exhibit strong stabilization via an added cost functional.*

### 3.2. Binomial Input

Now, let us move on to functionals of binomial point processes. Suppose that for n≥2, $\xi_{n}$ is a binomial point process guided by *Q*, and $F_{n}\in L_{\xi_{n}}^{2}\left(X\right)$ is a strongly stabilizing functional satisfying Assumptions 2–4.

**Theorem** **2** (Normal approximation under binomial input)**.** *Assume that $F_{n}$ is strongly stabilizing with stabilization radius satisfying Assumptions 2–4. In that case, there is a constant $C_{0}^{\prime }>0$, based only on the constants in those assumptions, such that for all n≥2,*

(12) $$d_{K}\left(\frac{F_{n}-E\left[F_{n}\right]}{\sqrt{\mathrm{Var}(F_{n})}},N\right)\leq C_{0}^{\prime }\left(\frac{\Theta_{K,n}^{1/2}}{\mathrm{Var}(F_{n})}+\frac{\Theta_{K,n}}{\mathrm{Var}{\left(F_{n}\right)}^{3/2}}+\frac{\Theta_{K,n}+\Theta_{K,n}^{3/2}}{\mathrm{Var}{\left(F_{n}\right)}^{2}}\right),$$

*where*

$$\Theta_{K,n}:=n\int_{X}\exp\left(-\frac{C_{4}\left(p-4\right)}{4p}\;{\left(d_{n}\left(x,K\right)\right)}^{2}\right)\;Q\left(dx\right)$$

*and $d_{n}\left(x,K\right):=\inf_{z\in K}n^{1/\beta_{Q}}d\left(x,z\right)$. Here, $\beta_{Q}$ expresses the metric growth parameter of the base measure Q, as described in Assumption 1.*

#### Interpretation of $\Theta_{K,s}$ and $\Theta_{K,n}$

Quantities $\Theta_{K,s}$ and $\Theta_{K,n}$ represent the exponential decay of tail probabilities as a function of stabilization radii and insertion cost magnitudes. Informally, we measure how quickly the function has become insensitive to points away from the corresponding set *K*. As these terms become uniformly bounded in variance, the resulting Kolmogorov bounds display the optimal rates $s^{-1/2}$ or $n^{-1/2}$.

**Remark** **5.** *The binomial limit in Theorem 2 corresponds to the Poisson case, although it has slightly different exponents because there is no exact binomial analogue of the second-order Poincaré inequality that exists in Poisson spaces; see [17] for the corresponding Berry–Esseen bounds.*

**Corollary** **1** (Optimal binomial convergence rate)**.** *We assume the setting of Theorem 2 and suppose that C>0 is a constant, so that*

(13) $$\underset{n\geq 1}{\sup}\frac{\Theta_{K,n}}{\mathrm{Var}(F_{n})}\leq C.$$

*Then, there is a constant $C_{0}^{\prime \prime }>0$, which depends only on C and the assumption constants, such that for all n≥2,*

$$d_{K}\left(\frac{F_{n}-E\left[F_{n}\right]}{\sqrt{\mathrm{Var}(F_{n})}},N\right)\leq \frac{C_{0}^{\prime \prime }}{\sqrt{\mathrm{Var}(F_{n})}}.$$

*Particularly, as $Var(F_{n})$ increases at least linearly with n, this gives the normal approximation rate of $O(n^{-1/2})$. A similar result holds for the Poisson model under similar stabilization and moment conditions. Particularly, if $\mathrm{Var}(F_{s})$ grows linearly with s, then Theorem 1 shows that the convergence rate is of the order $O(s^{-1/2})$.*

Theorems 1 and 2 summarize the general Poisson and binomial limits in Table 1.

Where

(14) $$R\left(\Theta_{K,n},\mathrm{Var}\left(F_{n}\right)\right)=\frac{\Theta_{K,n}^{1/2}}{\mathrm{Var}(F_{n})}+\frac{\Theta_{K,n}}{\mathrm{Var}{\left(F_{n}\right)}^{3/2}}+\frac{\Theta_{K,n}+\Theta_{K,n}^{3/2}}{\mathrm{Var}{\left(F_{n}\right)}^{2}}.$$

The constants $C^{*}$ and $C_{0}^{\prime }$ are only dependent on structural assumptions and moment parameters, as in Theorem 2.

## 4. Applications and Simulation Strategy

Now, we will explain the scope of Theorems 1 and 2 using several functionals of practical interest, focusing particularly on Tsallis entropy estimators derived from nearest neighbor distances. We will also summarize a simulation strategy for verifying theoretical bounds in concrete settings.

### 4.1. Tsallis Entropy Estimators Based on Nearest Neighbors

Consider *f* as a probability density function on $R^{d}$. For α≠1, the α-th-order Tsallis entropy is defined as follows:

(15) $$T_{\alpha }\left(f\right)=\frac{1}{1-\alpha }\left(\int_{R^{d}}f{\left(x\right)}^{\alpha }\;dx-1\right),\;\alpha \neq 1.$$

which is recovered by Shannon entropy as α→1 [1,3]. Given that X∼f, the integral term can be written as:

$$\int_{R^{d}}f{\left(x\right)}^{\alpha }\;dx=E\left[f{\left(X\right)}^{\alpha -1}\right].$$

Therefore, estimating $T_{\alpha }\left(f\right)$ can be reduced to estimating the expected value of $f{\left(X\right)}^{\alpha -1}$. An example point $X_{i}$ yields the *k*-nearest neighbor estimation of the density as follows:   

$${\hat{f}}_{k}\left(X_{i}\right)=\frac{k}{\left(n-1\right)\omega_{d}\rho_{k,i}^{d}},$$

where $\rho_{k,i}$ represents the distance from $X_{i}$ to its *k*-th NN between the remaining observations, and $\omega_{d}$ denotes the volume of the unit sphere in $R^{d}$. By substituting ${\hat{f}}_{k}\left(X_{i}\right)$ for $f{\left(X_{i}\right)}^{\alpha -1}$ and taking the average over the sample, we obtain the estimator for the nearest neighbor Tsallis estimator:

$${\hat{T}}_{\alpha ,n}=\frac{1}{1-\alpha }\left[\frac{1}{n}\sum_{i=1}^{n}{\left(\frac{k}{\left(n-1\right)\omega_{d}\rho_{k,i}^{d}}\right)}^{\alpha -1}-1\right].$$

We obtain the Poisson version ${\hat{T}}_{\alpha ,s}$ in a similar manner by replacing n−1 with the density parameter *s* and summing over the points $P_{s}$. Consider a Poisson process $P_{s}$ on $X\subseteq R^{d}$ with density sQ. Given a constant integer k≥1, let $\rho_{k}\left(x\right)$ denote the distance between $x\in P_{s}$ and its *k*-NN in $P_{s}$, and let $\omega_{d}$ be the volume of the unit ball in $R^{d}$. Assume

$$N_{s}:=\left|P_{s}\right|,\;N_{s}^{+}:=\max\left\{N_{s},1\right\}.$$

We define the normalized nearest-neighbor Tsallis estimator for the Poisson input as

$${\hat{T}}_{\alpha ,s}\left(P_{s}\right)=\frac{1}{1-\alpha }\left[\frac{1}{N_{s}^{+}}\sum_{x\in P_{s}}{\left(\frac{k}{s\omega_{d}\rho_{k}{\left(x,P_{s}\right)}^{d}}\right)}^{\alpha -1}-1\right].$$

Note that $N_{s}^{+}$ is used solely to avoid division by zero when $N_{s}=0$. If Q(S)=1, then $N_{s}∼\mathrm{Poisson}\left(s\right)$, and hence

$$P\left(N_{s}=0\right)=e^{-s},$$

so the change is exponentially negligible. The corresponding estimator for the binomial input is

$${\hat{T}}_{\alpha ,n}\left(\xi_{n}\right)=\frac{1}{1-\alpha }\left[\frac{1}{n}\sum_{i=1}^{n}{\left(\frac{k}{\left(n-1\right)\omega_{d}\rho_{k,i}^{d}}\right)}^{\alpha -1}-1\right],$$

where $\rho_{k,i}$ is the distance from $X_{i}$ to its *k*-th nearest neighbor from the remaining sample points.

#### 4.1.1. Bias and Consistency

The estimator exhibits the usual sources of bias associated with *k*-NN surrogate estimators. Among these are finite-sample bias in the local density estimate, boundary effects near the support of *f*, and the sensitivity of the power transformation $u\mapsto u^{\alpha -1}$. Given standard smoothness assumptions on *f*, a compact support, and a bounded density away from zero and infinity, the *k*-NN density estimator is locally consistent as $k=k_{n}\to \infty$ and $k_{n}/n\to 0$. Therefore, plugging in the estimate of $E[f{\left(X\right)}^{\alpha -1}]$ leads to a consistent result after controlling for boundary and moment terms. In this paper, we have developed a stabilization-based normal approximation for fixed *k* and fixed entropy index α. Thus, the goal is not to optimize the bias but to describe the fluctuation behavior of the corresponding local *k*-NN functional.

#### 4.1.2. Relation to Existing *k*-NN Entropy Estimators

Our proposed estimator is part of the family of nearest-neighbor pseudo- entropy estimators. Structurally, it is related to the Kozachenko–Leonenko estimator for Shannon entropy and the *k*-NN estimators for Rényi entropy, but it differs due to the Tsallis power transformation and ${\left(1-\alpha \right)}^{-1}$. As α→1, the Tsallis functional approaches Shannon entropy, while values of α≠1 alter the estimator’s sensitivity to low-density and high-density regions. In comparison to Tsallis goodness-of-fit estimators fully calibrated via simulation, this study focuses on the stabilization and normal approximation behavior of the underlying local *k*-NN functional. Hence, the originality lies not in the introduction of a completely different density estimator, but in the verification of the stabilization and moment conditions required to achieve explicit Gaussian approximation bounds for the Tsallis *k*-NN statistic.

#### 4.1.3. Choice of the Entropy Index α

In this theoretical analysis, α is treated not as an unknown parameter of the underlying distribution, but as a fixed entropy index. For this reason, normal approximation results are presented based on a predetermined value of α in the interval α∈(0,∞)∖{1}, and the constants at the limits may depend on this fixed value of α. From a statistical perspective, different choices of α highlight different parts of the distribution: values below and above 1 may change the estimator’s sensitivity to tail behavior and local concentration. For this reason, in empirical applications, α is determined through a sensitivity analysis on a finite grid, by minimizing the empirical mean squared error criterion when a reference model is provided, or by selecting the value that yields a stable goodness-of-fit calibration across Monte Carlo repetitions. An entirely model-based estimation theory for α goes beyond the scope of this paper; our goal here is to derive the normal approximation limits based on stabilization for each fixed acceptable entropy index.

We present a theorem that establishes the stabilization and moment properties justifying the application of the general normal approximation results to the Tsallis *k*-NN estimator.

**Proposition** **2** (Verification of stabilization and moment conditions for the *k*-NN Tsallis score)**.** *Suppose that Q satisfies a density f with respect to a compact subset $S\subset R^{d}$ and that there exist constants 0<m<M<∞ such that*

m≤f(x)≤M,x∈S.

*Suppose k≥1 is fixed and let α∈(0,∞)∖{1} be fixed. For $x\in P_{s}$, we define the local k-NN Tsallis score by*

$$\psi_{\alpha ,s}\left(x,P_{s}\right)={\left(\frac{k}{s\omega_{d}\rho_{k}{\left(x,P_{s}\right)}^{d}}\right)}^{\alpha -1},$$

*where $\rho_{k}\left(x,P_{s}\right)$ is defined as the distance from x to its k-NN in $P_{s}$. We assume that, for some p>4,*

$$p{\left(\alpha -1\right)}_{+}<k,\;{\left(\alpha -1\right)}_{+}:=\max\left\{\alpha -1,0\right\}.$$

*Then the score $\psi_{\alpha ,s}$, and therefore the associated estimator ${\hat{T}}_{\alpha ,s}$, stabilizes exponentially. Moreover, its first- and second-order add-one costs comply with a uniform $L^{p}$-moment bound of the following form:*

$$\underset{s\geq 1}{\sup}\underset{x,y\in S}{\sup}E\left[|\Delta_{x}{\hat{T}}_{\alpha ,s}{|}^{p}+{\left|\Delta_{x,y}{\hat{T}}_{\alpha ,s}\right|}^{p}\right]<\infty .$$

*We can say the same for the binomial estimator ${\hat{T}}_{\alpha ,n}$, where n replaces s.*

**Proof.**  Full proof is given in Appendix B. The treatment of the random Poisson denominator $N_{s}=\left|P_{s}\right|$ is also given in Appendix B.    □

**Theorem** **3** (Normal approximation for Tsallis entropy estimators)**.** *Assume the conditions of Proposition 2. Let there exist constants $C_{P},C_{B}>0$, depending purely on the structural constants k, d, p, and the fixed entropy index α, such that*

$$d_{K}\left(\frac{{\hat{T}}_{\alpha ,s}\left(P_{s}\right)-E\left[{\hat{T}}_{\alpha ,s}\left(P_{s}\right)\right]}{\sqrt{\mathrm{Var}({\hat{T}}_{\alpha ,s}\left(P_{s}\right))}},N\right)\leq \frac{C_{P}}{\sqrt{s}},$$

*and*

$$d_{K}\left(\frac{{\hat{T}}_{\alpha ,n}\left(\xi_{n}\right)-E\left[{\hat{T}}_{\alpha ,n}\left(\xi_{n}\right)\right]}{\sqrt{\mathrm{Var}({\hat{T}}_{\alpha ,n}\left(\xi_{n}\right))}},N\right)\leq \frac{C_{B}}{\sqrt{n}}.$$

**Remark** **6.** *Theorem 3 establishes a unified asymptotic normality consequence for a natural class of nearest-neighbor-based estimators of Tsallis entropy and supplements previous results on the consistency and mean-square approximation of such estimators in the context of goodness-of-fit [7,8]. Under similar stabilization assumptions, the $s^{-1/2}$ and $n^{-1/2}$ rates correspond to the behavior exhibited by Shannon and Rényi entropy estimators and are thus classical central limit scaling classical rates. Thus, the current finding presents a framework similar to the Rényi entropy estimation proposed in [9], but it is supported by stabilization techniques and Poisson/binomial estimation methods.*

### 4.2. Weighted *k*-NN Shannon Entropy

To complete, let us briefly recall the weighted *k*-NN estimator of Shannon entropy recommended in [6]. Assume that $X_{1},\ldots ,X_{n}$ are independent and identically distributed with density *q* on $R^{d}$. Then the Shannon entropy is

(16) $$H\left(q\right)=-\int_{R^{d}}q\left(x\right)\log q\left(x\right)\;dx.$$

Let $\rho_{j,i}$ denote the distance from $X_{i}$ to its *j*-th nearest neighbor in the set $\left\{X_{1},\ldots ,X_{n}\right\}\setminus \left\{X_{i}\right\}$. Suppose that $\omega_{d}$ is the volume of the unit ball in $R^{d}$ and Ψ is the digamma function. We obtain the estimator for weights ${\left\{w_{j}\right\}}_{j=1}^{k}$ satisfying the condition $\sum_{j=1}^{k}w_{j}=1$

(17) $$F_{n}^{\mathrm{SE}}\left(\xi_{n}\right)=\frac{1}{n}\sum_{i=1}^{n}\sum_{j=1}^{k}w_{j}\log\left(\frac{\left(n-1\right)\omega_{d}\rho_{j,i}^{d}}{e^{\Psi (j)}}\right)$$

which has appropriate bias and variance properties under mild regularity conditions.

**Theorem** **4** (Normal approximation for weighted *k*-NN Shannon estimator)**.** *Given Assumptions 2–4 and the conditions of regularity from [6] about q and the weights $\left\{w_{j}\right\}$, let there be constants $C_{0},\tau >0$ that, based only on these conditions, are such that for all sufficiently large n,*

(18) $$d_{K}\left(\frac{F_{n}^{\mathrm{SE}}\left(\xi_{n}\right)-H\left(q\right)}{\sqrt{\mathrm{Var}(F_{n}^{\mathrm{SE}}\left(\xi_{n}\right))}},N\right)\leq C_{0}{\left(\frac{k}{n}\right)}^{\tau }.$$

### 4.3. Geometric Functionals: Euler Characteristic and MST

As a geometric example, suppose we consider a binomial point process $\xi_{n}$ on ${\left[0,1\right]}^{d}$ having density *q*, which is bounded away from zero and infinity on ${\left[0,1\right]}^{d}$. Denote the Vietoris-Rips or Čech complex with scale r>0 by $K_{r}$ and write the Euler characteristic as χ(K). We define

(19) $$F_{n}^{\mathrm{EC}}\left(P_{n}\right)=\chi \left(K_{r}\left(n^{1/d}P_{n}\right)\right),\;F_{n}^{\mathrm{EC}}\left(\xi_{n}\right)=\chi \left(K_{r}\left(n^{1/d}\xi_{n}\right)\right).$$

**Theorem** **5** (Normal approach for the Euler characteristic)**.** *Assume that Assumptions 2–4 apply to the corresponding functionals of $P_{n}$ and $\xi_{n}$. Then there exists a constant $C_{\mathrm{EC}}>0$ with the following property:*

(20) $$d_{K}\left(\frac{F_{n}^{\mathrm{EC}}\left(\eta_{n}\right)-E\left[F_{n}^{\mathrm{EC}}\left(\eta_{n}\right)\right]}{\sqrt{\mathrm{Var}(F_{n}^{\mathrm{EC}}\left(\eta_{n}\right))}},N\right)\leq \frac{C_{\mathrm{EC}}}{\sqrt{n}},$$

*where $\eta_{n}$ denotes either $P_{n}$ or $\xi_{n}$.*

To find the normal approach rates for minimal spanning trees, let $V\subset R^{d}$ be a finite set of points and let M(V) represent the total length of the minimal spanning tree on *V*. Given that *V* is generated by a Poisson point process with intensity measure nQ, stabilization arguments and cost function techniques provide the following normal approach rates.

$$d_{K}\left(\frac{F_{n}^{\mathrm{MST}}-E\left[F_{n}^{\mathrm{MST}}\right]}{\sqrt{\mathrm{Var}(F_{n}^{\mathrm{MST}})}},N\right)\leq \left\{\begin{array}{cc} C_{\mathrm{MST}}\;n^{-\gamma_{1}}, & d=2,\\ C_{\mathrm{MST}}\;{\left(\log n\right)}^{-\gamma_{2}}, & d\geq 3, \end{array}\right.$$

for appropriate constants $C_{\mathrm{MST}},\gamma_{1},\gamma_{2}>0$ based on the basic distribution and dimension; see [10,14] for corresponding results.

We highlight the different rates achieved for the main functionals discussed in Theorems 1–5. We have summarized the corresponding Kolmogorov estimates in Table 2.

### 4.4. Monte Carlo Simulation Protocol

To increase the transparency and reproducibility of the numerical component, this paper describes the Monte Carlo protocol used to evaluate the finite-sample behavior of the stabilization-based normal approximation results. The key elements of the design have been outlined in Table 3, while the computational steps are detailed in Algorithm 1.
**Algorithm 1** Monte Carlo method for empirical normal approximation**Require:** Distributional scenario, dimension *d*, sample size *n*, entropy index α, nearest-neighbor parameter *k*, number of replications *B* **Ensure:** Empirical mean, empirical variance, standardized scores, and empirical Kolmogorov distance
  1:**for** b=1,…,B **do**  2:       Generate an independent sample$X_{1}^{\left(b\right)},\ldots ,X_{n}^{\left(b\right)}$
from the specified distribution.  3:       Compute the *k*-nearest-neighbor distances$\rho_{k,i}^{\left(b\right)},\;i=1,\ldots ,n.$  4:       Compute the Tsallis *k*-NN estimator${\hat{T}}_{\alpha ,n}^{\left(b\right)}.$When required, compute the corresponding weighted Shannon estimator.  5:**end for**  6:Estimate the Monte Carlo mean:${\bar{T}}_{\alpha ,n}=\frac{1}{B}\sum_{b=1}^{B}{\hat{T}}_{\alpha ,n}^{\left(b\right)}.$  7:Estimate the Monte Carlo variance:$\hat{\mathrm{Var}}\left({\hat{T}}_{\alpha ,n}\right)=\frac{1}{B-1}\sum_{b=1}^{B}{\left({\hat{T}}_{\alpha ,n}^{\left(b\right)}-{\bar{T}}_{\alpha ,n}\right)}^{2}.$  8:**for** b=1,…,B **do**  9:       Standardize the estimator:$Z_{n}^{\left(b\right)}=\frac{{\hat{T}}_{\alpha ,n}^{\left(b\right)}-{\bar{T}}_{\alpha ,n}}{\sqrt{\hat{\mathrm{Var}}\left({\hat{T}}_{\alpha ,n}\right)}}.$ 10:**end for** 11:Compute the empirical Kolmogorov distance:${\hat{d}}_{K}=\underset{t\in R}{\sup}\left|{\hat{F}}_{Z_{n}}\left(t\right)-\Phi \left(t\right)\right|,$
where ${\hat{F}}_{Z_{n}}$ represents the empirical distribution function of the standardized scores and Φ is the standard normal distribution function. 12:**return** ${\bar{T}}_{\alpha ,n}$, $\hat{\mathrm{Var}}\left({\hat{T}}_{\alpha ,n}\right)$, $Z_{n}^{\left(1\right)},\ldots ,Z_{n}^{\left(B\right)}$, and ${\hat{d}}_{K}$.

**Figure 1:**
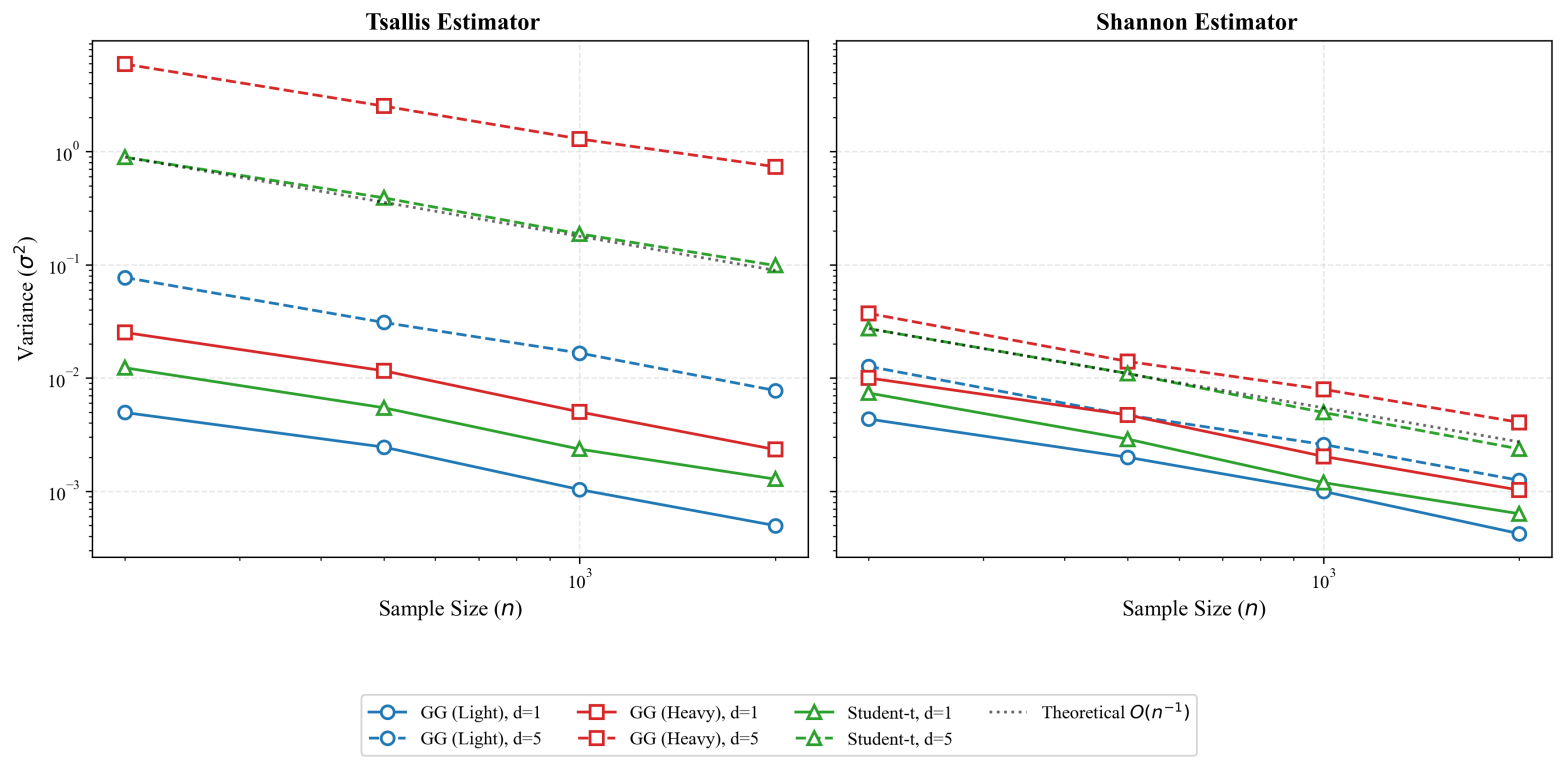
Empirical variance for the Tsallis and the weighted Shannon *k*-NN entropy estimators, as a function of sample size *n* on a log-log scale. The results are based on B=300 Monte Carlo replications per setup. You can see a grey dotted reference line with slope −1 that matches the theoretical variance scaling $\mathrm{Var}\left({\hat{T}}_{\alpha ,n}\right)=O\left(n^{-1}\right)$. In the panels, they juxtapose light-tailed generalized Gaussian, then heavy-tailed generalized Gaussian, plus Student-*t* cases, across a few chosen dimensions.

**Figure 2:**
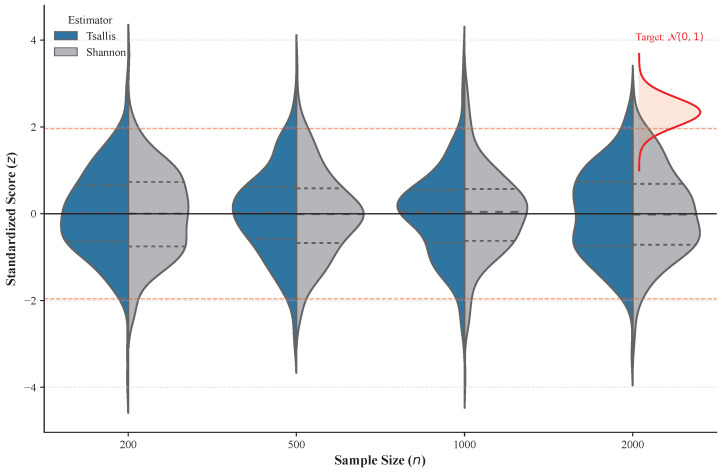
Violin plots of the standardized Tsallis and weighted Shannon *k*-NN entropy estimators for the bivariate generalized Gaussian model with shape parameter β=2.5. From each sample size n∈{200,500,1000,2000}, standardized scores are calculated from B=300 Monte Carlo simulations using the empirical Monte Carlo mean and variance. The dashed horizontal lines represent the ±1.96 standard normal quantiles. Increasing symmetry and concentration around zero suggest improved alignment with the Gaussian approximation.

We implemented the simulation described in Table 3 using the Monte Carlo workflow outlined in Algorithm 1. We recorded the empirical variance, scaled variance $n\hat{\mathrm{Var}}$, empirical Kolmogorov distance ${\hat{d}}_{K}$, and standardized scores for each configuration. As the paper’s main contribution is theoretical, the simulation protocol has been used as an illustrative diagnostic of variance scaling and approximate Gaussianity rather than as a separate empirical study. We interpret the graphical summaries in Figure 1 and Figure 2 in accordance with this protocol. In a typical setup, dimensions d∈{1,2,5}, sample sizes n∈{200,500,1000,2000}, and various fixed values of the entropy index α∈{0.8,1.0,1.2} are considered. Under the null hypothesis, simulations can be performed from multivariate generalized Gaussian distributions with different shape parameters. For each configuration, Tsallis, Shannon, and Rényi entropy estimators can be calculated, their empirical means and variances can be estimated, and the standardized estimator can be compared to the Gaussian benchmark across Monte Carlo replications to approximate the Kolmogorov distance to the normal distribution.

The main empirical variables of interest are:

- Bias and mean squared error of the Tsallis and Rényi entropy estimators for various n,d,α;
- Empirical variance and its scaled value in *n*;
- Empirical Kolmogorov distance between the normalized estimators and a normal distribution;
- Empirical size and power of Tsallis- and Rényi-based goodness-of-fit tests developed from the estimators.

Figure 1 displays the empirical variance of the Tsallis and weighted Shannon *k*-NN entropy estimators as a function of sample size *n* for several comparison distributions and sizes on a double logarithmic scale. In all scenarios examined, the variance is approximately linearly decreasing with logn. It has a slope close to −1, consistent with the theoretical estimate $\mathrm{Var}\left(F_{n}\right)=O\left(n^{-1}\right)$ suggested by the central limit theorems in Section 3. The alignment with the reference $O(n^{-1})$ line is particularly evident for medium and large sample sizes. It is robust with respect to changes in the underlying distribution (light-tailed generalized Gaussian, heavy-tailed generalized Gaussian, and Student-*t*) and dimension (d=1 and d=5). The Tsallis estimator exhibits slightly higher variance than the Shannon estimator in heavy-tailed and high-dimensional settings. However, both estimators have the same asymptotic slope, suggesting that the stabilization-based normal approximations capture the dominant scaling behavior of the fluctuation magnitude. These empirical findings support the optimality of the $n^{-1/2}$ rates at our Kolmogorov limits and show that theoretical results remain informative even at moderately high dimensions and under significant deviations from the Gaussian distribution.

Figure 2 supplies a qualitative evaluation of the asymptotic normality of the Tsallis and weighted Shannon *k*-NN entropy estimators. We generate n∈{200,500,1000,2000} observations for a light-tailed bivariate generalized Gaussian distribution (d=2, shape parameter β=2.5) and computed over 300 Monte Carlo replications, which were found to provide stable estimates while keeping computational costs moderate. Monte Carlo replications. We use the following formula to standardize the resulting estimates $\hat{H}$ for each *n*:

(21) $$Z_{n}=\frac{\hat{H}-E\left[\hat{H}\right]}{\sqrt{\mathrm{Var}(\hat{H})}},$$

ensuring that any deviation from the standard normal distribution is reflected in the shape of the standardized sample.

The violin plots in Figure 2 show how the empirical distributions of the standardized Tsallis and Shannon estimators change as the sample size increases. The fact that the distribution’s mean is at zero and the reference quantile alignment indicate that the normal approximation holds for every *n* value examined by the researchers. The n=200 sample shows that both estimators deviate from Gaussianity due to their asymmetrical distributions and additional tail weight. The distribution shapes develop symmetric patterns while their distribution mass begins to concentrate at the origin. The Tsallis estimator displays a dispersion pattern that is very similar to that of the Shannon estimator at all sample sizes considered, indicating that the use of a generalized entropy index (here α=1.2) does not lead to slower convergence or markedly heavier tails. The $O(n^{-1/2})$ convergence rate with theoretical variance scaling indicates that higher *n* values lead to a decrease in variability. Empirical results supporting the theoretical findings indicate that stabilization-based normal approximations accurately characterize the distributional behavior of estimators. The visual diagnostics confirm the quantitative Kolmogorov distance results, which match the central limit theorems established in Section 3 because both estimators reach asymptotic normality under the stabilization and moment assumptions established in this study.

## 5. Proofs of Main Results

The section provides proofs of all main normal approximation results documented in Section 3. The study demonstrates how adaptive add-one cost decompositions interact with second-order Poincaré-type inequalities.

### 5.1. Poisson Case

The proof of Theorem 1 requires two components, which include the second-order Poincaré inequality for Poisson functionals proved by [15] together with an adaptive decomposition of the increment operators. For convenience, we recall a simplified version of the main inequality.

**Theorem** **6** (Last–Peccati–Schulte [15])**.** *Suppose F is a square-integrable function of the Poisson point process P(λ) with intensity measure λ, satisfying*

(22) $$E\left[F^{2}\right]<\infty \;\mathrm{and}\;E\left[\int_{X}{\left(\Delta_{x}F\right)}^{2}\;\lambda \left(dx\right)\right]<\infty .$$

*Then*

$$d_{K}\left(\frac{F-E[F]}{\sqrt{\mathrm{Var}(F)}},N\right)\leq \sum_{i=1}^{6}\gamma_{i},$$

*where $\gamma_{1},\ldots ,\gamma_{6}$ denote explicit integrals involving the first and second derivatives of F.*

**Proof** **of Theorem 1.** We obtain the proof by applying the second-order Poincaré inequality for Poisson functionals with respect to the localized add-one cost decomposition. Assume that $\sigma_{F}^{2}=\mathrm{Var}\left(F\right)>0.$ Let us write, for each x∈X,

$$\Delta_{x}F=\left(\Delta_{x}F-\Delta_{x}F\left(A_{x}\right)\right)+\Delta_{x}F\left(A_{x}\right).$$

Let the first term measure the localization error, while the second term is the localized add-one cost. Likewise, let the second-order increment $\Delta_{x,y}F$ be decomposed into localized and residual parts based on the same family of localization sets $A_{x}$ and $A_{y}$. The application of the Poisson second-order Poincaré inequality to *F* yields six Malliavin–Stein error terms that involve products of first- and second-order increments. The localization decomposition separates these terms into contributions governed by

$$b_{1}\left(x,A_{x}\right)=E{\left|\Delta_{x}F-\Delta_{x}F\left(A_{x}\right)\right|}^{4},\;b_{2}\left(x,A_{x}\right)=E{\left|\Delta_{x}F\left(A_{x}\right)\right|}^{4},$$

and analogous quantities that involve $\Delta_{x,y}F$. According to Hölder’s inequality and the assumed $L^{p}$-moment bounds, each expression is bounded by a normalized integral that involves $\Delta_{x}F$, $\Delta_{x,y}F$, and the intensity measure $\lambda_{s}$. These normalized integrals represent precisely the quantities $\Gamma_{1},\ldots ,\Gamma_{6}$, listed explicitly in Appendix A. We combine the six resulting estimates and absorb universal constants into $C^{*}$ to produce

$$d_{K}\left(\frac{F-E[F]}{\sqrt{\mathrm{Var}(F)}},N\right)\leq C^{*}\sum_{i=1}^{6}\Gamma_{i}.$$

Given the spatial localization and moment assumptions, the terms $\Gamma_{i}$ approach zero as s→∞, which establishes the asserted normal approximation bound. □

### 5.2. Binomial Case

Theorem 6 does not have its exact equivalent for the binomial setting, yet [17] proved related inequalities. Theorem 2 results from applying their proof methods to our cost-operator framework. The process for this method follows the same steps as the Poisson method, except that discrete sums replace integrals and suitable combinatorial factors are used.

**Proof** **of Theorem 2.** The proof derives from the binomial normal approximations for stabilizing functionals, integrated with the spatial localization and moment assumptions discussed in Section 2. Given x∈X, the add-one cost $\Delta_{x}F_{n}$ decomposes into a localized term with a residual term of the form

$$\Delta_{x}F_{n}=\left(\Delta_{x}F_{n}-\Delta_{x}F_{n}\left(A_{x}\right)\right)+\Delta_{x}F_{n}\left(A_{x}\right).$$

We apply the same decomposition to the second-order add-one cost $\Delta_{x,y}F_{n}$. We can express the residual terms in the form of a sum of terms with exponential spatial localization, while the localized terms are governed by the uniform $L^{p}$-moment assumption. The binomial Berry–Esseen estimate then yields three types of contributions: a first-order localization term, a second-order interaction term, and a higher-order remaining term. By applying Hölder’s inequality and the spatial decay assumption, these contributions become bounded, respectively, by

$$\frac{\Theta_{K,n}^{1/2}}{\mathrm{Var}(F_{n})},\;\frac{\Theta_{K,n}}{\mathrm{Var}{\left(F_{n}\right)}^{3/2}},\;\frac{\Theta_{K,n}+\Theta_{K,n}^{3/2}}{\mathrm{Var}{\left(F_{n}\right)}^{2}}.$$

Here $\Theta_{K,n}$ denotes the effective spatial localization quantity described in Theorem 2. Applying these bounds yields

$$d_{K}\left(\frac{F_{n}-E\left[F_{n}\right]}{\sqrt{\mathrm{Var}(F_{n})}},N\right)\leq C_{0}^{\prime }\left(\frac{\Theta_{K,n}^{1/2}}{\mathrm{Var}(F_{n})}+\frac{\Theta_{K,n}}{\mathrm{Var}{\left(F_{n}\right)}^{3/2}}+\frac{\Theta_{K,n}+\Theta_{K,n}^{3/2}}{\mathrm{Var}{\left(F_{n}\right)}^{2}}\right),$$

where $C_{0}^{\prime }$ is only based on the constants in the stabilization, spatial decay, and moment assumptions. □

### 5.3. Proof of Corollary 1

The corollary follows by choosing $A_{x}=X$, which simplifies the decomposition so that $b_{1}\equiv 0$ and the main contribution comes from $b_{2}$. The assumptions imply that $\Theta_{K,n}$ stays under uniform control when compared to $\mathrm{Var}(F_{n})$ and Theorem 2 establishes a bound which reduces to the value of $\mathrm{Var}{\left(F_{n}\right)}^{-1/2}$. The details are similar to those of the Poisson case examined in [16], and therefore we will not include them.

## 6. Conclusions

The research establishes general stabilization-based processes that researchers can use to prove central limit theorems, providing specific Kolmogorov convergence rates for all functions derived from Poisson and binomial point processes. This method provides first- and second-order growth control through the systematic application of adaptable addition cost operators, eliminating the need for detailed stabilization radius calculations and explicit point-function decompositions.

The framework provides optimal-order normal approximation results for nearest-neighbor Tsallis entropy estimators based on natural regularity and tail and moment conditions applied to the underlying metric measure space. The same methodology recovers and extends existing normal approximation results for weighted *k*-NN Shannon entropy estimators, Euler characteristics of random geometric complexes, and minimal spanning tree functionals, highlighting the unifying role of stabilization techniques in geometric probability and information-theoretic estimation.

The results presented here suggest several directions for further research. This work can be extended by testing Tsallis and related entropy estimators in non-Euclidean spaces, such as manifolds and graphs, as these spaces possess unique geometric properties that affect nearest-neighbor relations and stabilization methods. It can be adapted to select the *k*-NN parameter. It will investigate how data-driven approaches perform in high-dimensional settings, examining their impact on bias and variance during stabilization. The current standard approach should be better combined with entropy-based goodness-of-fit tests and dependency modelling to develop tests and estimators that exhibit strong asymptotic performance in complex, high-dimensional contexts while preserving reliability.

## Figures and Tables

**Table 1:** Asymptotic normality and Kolmogorov estimates for general stabilizing processes under the assumptions of Section 2.

Functional	Input Process	Rate at $d_{K}$
*F*	Poisson $P_{s}$	$C^{*}\;\sum_{i=1}^{6}\Gamma_{i}$
$F_{n}$	Binomial $\xi_{n}$	$C_{0}^{\prime }\;R\left(\Theta_{K,n},\mathrm{Var}\left(F_{n}\right)\right)$

**Table 2:** The asymptotic normality and Kolmogorov rates for a set of entropy-related and geometric functionals under the stabilization conditions of Section 2.

Functional	Input Process	Target Quantity	Rate in $d_{K}$
Tsallis *k*-NN estimator $F_{s}^{\left(\alpha \right)}$	Poisson $P_{s}$	Tsallis entropy	$C_{P}\;s^{-1/2}$
Tsallis *k*-NN estimator $F_{n}^{\left(\alpha \right)}$	Binomial $\xi_{n}$	Tsallis entropy	$C_{B}\;n^{-1/2}$
Weighted *k*-NN estimator $F_{n}^{\mathrm{SE}}$	Binomial $\xi_{n}$	Shannon entropy	$C_{0}{\left(k/n\right)}^{\tau }$
Euler characteristic $F_{n}^{\mathrm{EC}}$	Poisson or binomial	Euler characteristic	$C_{\mathrm{EC}}\;n^{-1/2}$
MST total length $F_{n}^{\mathrm{MST}}$	Poisson $P_{n}$	MST length	$C_{\mathrm{MST}}n^{-\gamma_{1}}$ or $C_{\mathrm{MST}}{\left(\log n\right)}^{-\gamma_{2}}$

**Table 3:** Monte Carlo design utilized to evaluate the finite-sample normal estimation of the nearest-neighbor entropy estimators.

Component	Details
Input models	Binomial samples from generalized Gaussian and Student-*t* distributions; homogeneous Poisson samples on bounded subsets of $R^{d}$.
Dimensions	d∈{1,2,5}.
Sample sizes	n∈{200,500,1000,2000}.
Entropy indices	Fixed values α∈{0.8,1.0,1.2}, where α=1 is the Shannon limiting case.
Nearest-neighbor parameter	Fixed *k*, selected identically across sample sizes within a given scenario.
Monte Carlo simulations	B=300 replications for each configuration.
Reported quantities	Empirical mean, empirical variance, scaled variance $n\;\hat{\mathrm{Var}}$, empirical Kolmogorov distance, and standardized scores $Z_{n}$.
Purpose	The aim is to examine variance scaling, approximate Gaussianity, and the finite-sample behavior proposed by the stabilization-based normal approximation bounds.

## Data Availability

The code and figure outputs used for the asymptotic analysis of the Tsallis and Shannon entropy estimators are available at: https://github.com/mehmetsiddik/asymptotic-tsallis-entropy-estimators (accessed on 29 May 2026).

## Appendix A. Malliavin–Stein Quantities Used in the Poisson Bound

This appendix provides an explicit definition of the Malliavin–Stein measures used in Theorem 1. We follow the notation developed by Last, Peccati, and Schulte [15] for second-order Poincaré inequalities for Poisson functionals and the stabilization formulation used for geometric Poisson functionals. Given a Poisson point process $P_{s}$ on (X,F) with intensity measure $\lambda_{s}=sQ$, let $F=F(P_{s})$ be a square-integrable Poisson functional satisfying

$$\sigma_{s}^{2}:=\mathrm{Var}\left(F\right)>0.$$

Define the first- and second-order difference operators for x,y∈X by

$$D_{x}F:=F\left(P_{s}+\delta_{x}\right)-F\left(P_{s}\right),$$

and

$$D_{x,y}^{2}F:=F\left(P_{s}+\delta_{x}+\delta_{y}\right)-F\left(P_{s}+\delta_{x}\right)-F\left(P_{s}+\delta_{y}\right)+F\left(P_{s}\right).$$

It is assumed that F∈domD, meaning that

$$E\left[F^{2}\right]<\infty ,\;E\left[\int_{X}{\left(D_{x}F\right)}^{2}\;\lambda_{s}\left(dx\right)\right]<\infty .$$

To get the Kolmogorov bound, let us define the fifth-moment constants

$$c_{1,s}:=\underset{x\in X}{ess sup}E\left[|D_{x}{F|}^{5}\right],\;c_{2,s}:=\underset{x,y\in X}{ess sup}E\left[|D_{x,y}^{2}{F|}^{5}\right],$$

where the essential suprema are obtained with respect to $\lambda_{s}$ and $\lambda_{s}^{2}$, respectively. According to Assumption 4, these quantities are finite.

In Theorem 1, the six Malliavin–Stein error terms are described as follows:

(A1) $$\begin{array}{cc} \Gamma_{1,s}\left(F\right) & :=\frac{2{\left(c_{1,s}c_{2,s}\right)}^{1/5}}{\sigma_{s}^{2}}{\left[\int_{X^{3}}{\left\{P\left(D_{x_{1},x_{3}}^{2}F\neq 0\right)P\left(D_{x_{2},x_{3}}^{2}F\neq 0\right)\right\}}^{1/20}\;\lambda_{s}^{3}\left(d\left(x_{1},x_{2},x_{3}\right)\right)\right]}^{1/2}, \end{array}$$

(A2) $$\begin{array}{cc} \Gamma_{2,s}\left(F\right) & :=\frac{c_{2,s}^{2/5}}{\sigma_{s}^{2}}{\left[\int_{X^{3}}{\left\{P\left(D_{x_{1},x_{3}}^{2}F\neq 0\right)P\left(D_{x_{2},x_{3}}^{2}F\neq 0\right)\right\}}^{1/10}\;\lambda_{s}^{3}\left(d\left(x_{1},x_{2},x_{3}\right)\right)\right]}^{1/2}, \end{array}$$

(A3) $$\begin{array}{cc} \Gamma_{3,s}\left(F\right) & :=\frac{1}{\sigma_{s}^{3}}\int_{X}E\left[|D_{x}{F|}^{3}\right]\;\lambda_{s}\left(dx\right), \end{array}$$

(A4) $$\begin{array}{cc} \Gamma_{4,s}\left(F\right) & :=\frac{c_{1,s}^{3/5}\lambda_{s}\left(X\right)}{\sigma_{s}^{3}}+\frac{c_{1,s}^{4/5}\lambda_{s}{\left(X\right)}^{5/4}+2c_{1,s}^{4/5}\lambda_{s}{\left(X\right)}^{3/2}}{\sigma_{s}^{4}}, \end{array}$$

(A5) $$\begin{array}{cc} \Gamma_{5,s}\left(F\right) & :=\frac{c_{1,s}^{2/5}\lambda_{s}{\left(X\right)}^{1/2}}{\sigma_{s}^{2}}, \end{array}$$

(A6) $$\begin{array}{cc} \Gamma_{6,s}\left(F\right) & :=\frac{\sqrt{6}{\left(c_{1,s}c_{2,s}\right)}^{1/5}+\sqrt{3}c_{2,s}^{2/5}}{\sigma_{s}^{2}}{\left[\int_{X^{2}}P{\left(D_{x_{1},x_{2}}^{2}F\neq 0\right)}^{1/10}\;\lambda_{s}^{2}\left(d\left(x_{1},x_{2}\right)\right)\right]}^{1/2}. \end{array}$$

According to these definitions, the Kolmogorov limit used in Theorem 1 can be expressed as follows:

$$d_{K}\left(\frac{F-E[F]}{\sqrt{\mathrm{Var}(F)}},N\right)\leq \sum_{i=1}^{6}\Gamma_{i,s}\left(F\right).$$

The probabilities appearing in $\Gamma_{1,s}\left(F\right)$, $\Gamma_{2,s}\left(F\right)$, and $\Gamma_{6,s}\left(F\right)$ are measures of the second-order difference operators being nonzero. In the stabilization of geometric functionals, these probabilities are determined by the localization radius and diminish exponentially as the included points move farther apart in the rescaled metric. This provides the connection between the abstract Malliavin-Stein limit and the stabilization hypotheses employed in the main text.

## Appendix B. Detailed Proof of Proposition 2

For the nearest-neighbor Tsallis score, we offer details concerning stabilization and moment validation. Proof is provided for the Poisson distribution; for the binomial distribution, we apply the standard binomial analog using sample size *n* in replacement of the Poisson density parameter *s*.

Step 1: Local dependence of the *k*-nearest-neighbor score.

The local Tsallis score for $x\in P_{s}$ is given by:

$$\psi_{\alpha ,s}\left(x,P_{s}\right)={\left(\frac{k}{s\omega_{d}\rho_{k}{\left(x,P_{s}\right)}^{d}}\right)}^{\alpha -1},$$

where $\rho_{k}\left(x,P_{s}\right)$ denotes the distance from *x* to its *k*-th nearest neighbor within $P_{s}$. Thus, by the configuration of points inside the sphere, the value of $\psi_{\alpha ,s}\left(x,P_{s}\right)$ is given by:

$$B_{x}\left(\rho_{k}\left(x,P_{s}\right)\right).$$

As a result, if the outside configuration of a sphere including the *k*-nearest neighbors of *x* is modified, the score remains unchanged. This demonstrates the local dependence of the score.

Step 2: Exponential stabilization.

We take $R_{s}\left(x\right)$ as the stabilization radius for the point *x*. It is sufficient for the ball $B_{x}\left(R_{s}\left(x\right)\right)$ to include the points that determine the *k*-nearest-neighbor structure of *x*. For a compact support *S* under the lower density condition f≥m>0, for a sufficiently small r>0,

$$Q\left(B_{x}\left(r\right)\cap S\right)\geq c_{1}r^{d}.$$

Thus, for a Poisson point processes with density measure sQ,

$$P\left\{P_{s}\left(B_{x}\left(r\right)\cap S\right)<k\right\}\leq \sum_{j=0}^{k-1}\exp\left\{-sQ\left(B_{x}\left(r\right)\cap S\right)\right\}\frac{{\left(sQ\left(B_{x}\left(r\right)\cap S\right)\right)}^{j}}{j!}.$$

By using $Q\left(B_{x}\left(r\right)\cap S\right)\geq c_{1}r^{d}$, the right-hand side is restricted to the appropriate constants $C_{1},C_{2}>0$ such that

$$C_{1}\exp\left\{-C_{2}sr^{d}\right\}.$$

Alternatively, by setting $t=s^{1/d}r$,

$$P\left\{s^{1/d}R_{s}\left(x\right)>t\right\}\leq C_{1}\exp\left\{-C_{2}t^{d}\right\}.$$

Therefore, the stabilization radius exhibits an exponential tail in the rescaled metric. The same argument applies to the binomial point process by using the corresponding binomial lower-tail bound. Consequently, the *k*-nearest-neighbor Tsallis score stabilizes exponentially.

Step 3: Stabilization of the add-one costs.

Adding a point has a local cost; in fact, adding a point *z* affects only the scores of those points that have become *k* nearest neighbors of *z*. Given a fixed *k* and a fixed dimension *d*, the total number of points involved in this process is governed by a deterministic constant depending only on k,d. Thus, the first-order insertion cost can be written as a finite sum of local score differences. Likewise, a second-order addition cost contains only points simultaneously affected in the *k*-nearest-neighbor structure by both added points. Therefore, both $\Delta_{z}{\hat{T}}_{\alpha ,s}$ and $\Delta_{z,w}{\hat{T}}_{\alpha ,s}$ determine the configuration within a finite combination of stabilizing spheres.

Step 4: Uniform $L^{p}$-moment control.

The remaining task is to verify that the first- and second-order “addition” costs possess uniform finite *p*-th moments in the interval *s*. Due to the upper density bound f≤M, the local excess density can be uniformly controlled. The possible singular behavior originates from the small *k*-nearest-neighbor distances. The standard Poisson nearest-neighbor calculation for the *k*-nearest-neighbor volume yields finite negative moments when the following condition is satisfied:

$$p{\left(1-\alpha \right)}_{+}<k.$$

It guarantees that the power term appearing in the Tsallis score has a finite *p*-th moment when α<1. When α>1, the compactness of *S* and the exponential tail of the stabilization radius control the correspondingly positive powers of the nearest-neighbor distance.

We obtain the following by combining the finite local interaction property from Step 3 with the moment bound above:

$$\underset{s\geq 1}{\sup}\underset{z,w\in S}{\sup}E\left[|\Delta_{z}{\hat{T}}_{\alpha ,s}{|}^{p}+{\left|\Delta_{z,w}{\hat{T}}_{\alpha ,s}\right|}^{p}\right]<\infty .$$

Using the same argument, we can apply this to the binomial estimator ${\hat{T}}_{\alpha ,n}$, where *n* replaces *s*. Then, as claimed in Proposition 2, it has exponential stabilization and uniform $L^{p}$-moment control.

Step 5: Control of the random Poisson denominator.

It remains to explain the role of the random denominator in the Poisson version of the estimator. Suppose

$$N_{s}:=\left|P_{s}\right|,\;N_{s}^{+}:=\max\left\{N_{s},1\right\},$$

and let

$$S_{s}\left(P_{s}\right):=\sum_{x\in P_{s}}\psi_{\alpha ,s}\left(x,P_{s}\right),\;{\bar{S}}_{s}\left(P_{s}\right):=\frac{S_{s}\left(P_{s}\right)}{N_{s}^{+}}.$$

For Q(S) = 1, $N_{s}∼\mathrm{Poisson}\left(s\right)$. More generally, $N_{s}∼\mathrm{Poisson}\left(sQ\left(S\right)\right)$ if the support has a finite *Q*-measure. For the normalized case Q(S)=1, it follows that

$$P\left(N_{s}=0\right)=e^{-s},$$

and, for every fixed r>0,

$$E\left[{\left|\frac{N_{s}}{s}-1\right|}^{r}\right]=O\left(s^{-r/2}\right),\;E\left[{\left(N_{s}^{+}\right)}^{-r}\right]=O\left(s^{-r}\right).$$

In fact, we can get the second bound by splitting the expectation over $\left\{N_{s}\geq s/2\right\}$ and $\left\{N_{s}<s/2\right\}$, and applying the standard lower-tail concentration bound for Poisson random variables. Given an inserted point *z*, we have that the first-order add-one cost of the normalized sum satisfies

$$D_{z}{\bar{S}}_{s}=\frac{D_{z}S_{s}}{N_{s}+1}+S_{s}\left(\frac{1}{N_{s}+1}-\frac{1}{N_{s}+1}\right).$$

For the event $\left\{N_{s}\geq 1\right\}$, it can be written as

$$D_{z}{\bar{S}}_{s}=\frac{D_{z}S_{s}}{N_{s}+1}-\frac{S_{s}}{N_{s}\left(N_{s}+1\right)}.$$

We can see that the first term is determined by the same local stabilization method used for the unnormalized score, combined with the fact that $N_{s}+1≍s$ occurs with high probability. The second term is a solely normalizing contribution and is controlled by the Poisson distribution of $N_{s}$ around *s*, together with the moment bound for the local score sum $S_{s}$. The occurrence of $N_{s}=0$ provides only an exponentially small error of order $e^{-s}$.

The second-order add-one cost is dealt with in a similar manner by using another difference operator on the above expression. It consists of a combination of normalization expressions containing the following factors, obtained by dividing the local second-order score contributions by $N_{s}+2$:

$$\frac{1}{N_{s}+1},\;\frac{1}{\left(N_{s}+1\right)\left(N_{s}+2\right)},\;\frac{1}{N_{s}\left(N_{s}+1\right)}.$$

Based on the above Poisson concentration limits, all these factors have a $O(s^{-1})$ or $O(s^{-2})$ order of magnitude within $L^{r}$, excluding an exponentially small error. As a result, the random denominator does not change the stabilization radius, the uniform $L^{p}$-moment control, or the order of the normal approximation limit.
